# Anti-mosquito properties of *Pelargonium roseum* (Geraniaceae) and *Juniperus virginiana* (Cupressaceae) essential oils against dominant malaria vectors in Africa

**DOI:** 10.1186/s12936-022-04220-8

**Published:** 2022-07-14

**Authors:** Revocatus Yohana, Paulo S. Chisulumi, Winifrida Kidima, Azar Tahghighi, Naseh Maleki-Ravasan, Eliningaya J. Kweka

**Affiliations:** 1 Department of Zoology and Wildlife Conservation, College of Natural and Applied Sciences, University of Dar Es Salaam, Dar Es Salaam, Tanzania; 2 Laboratory of Medicinal Chemistry, Department of Clinical Research, Pasteur Institute of Iran, Tehran, Iran; 3 Department of Parasitology, Pasteur Institute of Iran, Tehran, Iran; 4 Department of Medical Parasitology and Entomology, School of Medicine, Catholic University of Health Sciences, Mwanza, Tanzania; 5 Tropical Pesticides Research Institute, Division of Livestock and Human Disease Vector Control Mosquito Section, Arusha, Tanzania

**Keywords:** Malaria, *Anopheles gambiae*, Essential oils, Geranium, Juniper, Larvicidal, Knockdown, Mortality

## Abstract

**Background:**

More than 90% of malaria cases occur in Africa where the disease is transmitted by *Anopheles gambiae* and *Anopheles arabiensis*. This study evaluated the anti-mosquito properties of *Juniperus virginiana* (JVO) and *Pelargonium roseum* (PRO) essential oils (EOs) against larvae and adults of *An. gambiae sensu lato (s.l.*) from East Africa in laboratory and semi-field conditions.

**Methods:**

EOs was extracted from the aerial green parts of Asian herbs by hydrodistillation. Their constituents were characterized by gas chromatography-mass spectrometry (GC-MS). Larvicidal activities of JVO, PRO, and PRO components [citronellol (CO), linalool (LO), and geraniol (GO)] were investigated against *An. gambiae sensu stricto* (*s.s*.). The percentage of knockdown effects and mortality rates of all oils were also evaluated in the adults of susceptible *An. gambiae s.s.* and permethrin-resistant *An. arabiensis*.

**Results:**

GC-MS analyses identified major constituents of JVO (sabinene, dl-limonene, β-myrcene, bornyl acetate, and terpinen-4-ol) and PRO (citronellol, citronellyl formate, L-menthone, linalool, and geraniol). Oils showed higher larvicidal activity in the laboratory than semi-field trials. The LC_50_ values for JVO/PRO were computed as 10.82–2.89/7.13–0.9 ppm and 10.75–9.06/13.63–8.98 ppm in laboratory and semi-field environments, respectively at exposure time of 24–72 h. The percentage of knockdown effects of the oils were also greater in *An. gambiae s.s.* than in *An. arabiensis*. Filter papers impregnated with JVO (100 ppm) and PRO (25 ppm) displayed 100% mortality rates for *An. gambiae s.s.* and 3.75% and 90% mortality rates, for *An. arabiensis* populations, respectively. Each component of CO, LO, and GO exhibited 98.13%, 97.81%, and 87.5%, respectively, and a mixture of the PRO components indicated 94.69% adult mortality to permethrin-resistant *An. arabiensis*.

**Conclusions:**

The findings of this study show that PRO and its main constituents, compared to JVO, have higher anti-mosquito properties in terms of larvicidal, knockdown, and mortality when applied against susceptible laboratory and resistant wild populations of *An. gambiae s.l.* Consequently, these oils have the potential for the development of new, efficient, safe, and affordable agents for mosquito control.

**Supplementary Information:**

The online version contains supplementary material available at 10.1186/s12936-022-04220-8.

## Background

Malaria is a serious tropical and sometimes life-threatening disease caused by *Plasmodium* parasites and spread by the infected bites of female mosquitoes (Diptera: Culicidae). According to the latest reports, an estimated 229 million cases of malaria and 409,000 deaths were recorded in 2019 worldwide [1]. Most malaria cases and deaths occur in sub-Saharan Africa (93%), followed by the WHO South-East Asia Region (3.4%) and the WHO Eastern Mediterranean Region (2.1%) [1].

In Africa, malaria is transmitted by nine sibling species of the *Anopheles gambiae* complex with diverse bionomics, among which *An. gambiae sensu stricto* (*s.s*.) and *Anopheles arabiensis* breed in freshwater. By virtue of the anthropophilic and endophilic characteristics, *An. gambiae s.s.* feeds and rests indoors [2, 3]. However, due to the application of chemical insecticides and repellents, its natural ecological feeding and resting behaviour have been shifted from indoors to outdoors [4, 5]. *Anopheles arabiensis*, unlike *An. gambiae s.s.*, has zoophilic tendencies and feeds on a range of mammalian hosts [6]. In Tanzania, like other African countries, malaria transmission is complicated owing to differences in vector species composition. *Anopheles gambiae s.s.* and *Anopheles funestus* are predominant vectors in humid coastal regions, whereas *An*. *arabiensis* serves as the main vector in arid interior regions [7, 8].

Targeting vector mosquitoes is a key tactic to combat mosquito-borne diseases and to prevent pathogen transmission [9]. Other strategies include larval source management, larviciding, and controlling adult mosquitoes by insecticides and repellents [10, 11]. The chemical insecticides used to control malaria-carrying mosquitoes belong to four main classes: organochlorines, organophosphates, carbamates, and pyrethroids [12]. A dozen of insecticides in the aforementioned classes are suggested for use in indoor residual spraying. However, only pyrethroids are now recommended to be employed in insecticide-treated bed nets [13].

Today, many commercial mosquitocides and repellents are recommended for use on humans and animals [14]. Despite the effectiveness of chemical pesticides in vector control, their prolonged and inappropriate application have led to environmental hazards, mammalian toxicity, and the emergence of resistant strains [15]. Therefore, interest in research and development of botanical pesticides has been intensified as they affect only target organisms and mostly do not damage valuable natural enemies, as well as provide residue-free nutrition and a safe environment [16–18].

The plant-derived essential oils (EOs) show a broad spectrum of activity against pest insects, including insecticidal, repellent, oviposition-deterrent, antifeedant, antivector, and growth regulatory activities [19, 20]. EOs, often called green pesticides, are volatile oils extracted from plants by different methods, including hydrodistillation. The components of the oils are secondary metabolites produced by aromatic plants [21]. These biopesticides have been regarded as an alternative to synthetic insecticides in public health sections, food industries, and agriculture and have been indicated to cause decreased pest population, health promotion, and increased food productions [21, 22]. They potently and rapidly influence the target and are degraded swiftly in the environment [23]. Therefore, it is reasonable to consider that the effects of the mentioned biopesticides on human health and environment are weaker than most pesticides.

*Pelargonium* species (Geraniaceae) are evergreen perennials genus of flowering plants with about 280 species, commonly known as pelargoniums, storksbills, or simply geraniums [24, 25]. They are resistant to heat and drought and have a global distribution in tropical, subtropical, and temperate climates [26]. *Pelargonium roseum* (PRO), a *Pelargonium* species, is cultivated for its beauty as an ornamental plant and also for its scent as an important ingredient in perfume, food, and beverage industries [27]. In traditional medicine, *Pelargonium* species have been applied to treat fevers, intestinal problems, wounds, respiratory ailments, kidney complaints, gastroenteritis, hemorrhage, neuralgia, throat infections, and other conditions [28, 29].

Junipers are coniferous trees and shrubs in the genus *Juniperus* (Cupressaceae). This genus with about 70 species is widely distributed throughout the northern hemisphere and has a global distribution as indigenous and nonindigenous plants [30]. Junipers are cultivated for timber, culinary use, EOs, and ethnic and herbal use [31]. Different parts of *Juniperus* are employed as stimulant, stomachic, carminative, antihelminthic, wound healing, antiseptic, antifungal, antirheumatic, expectorant, insecticide, and diuretic agents in traditional medicine [30, 32, 33]. *Juniperus virginiana*, commonly known as eastern red cedar, can withstand a variety of extreme climates and conditions. It is acknowledged for its aromatic odour, toxicity, and repellency to numerous species of insects, clothes moths [34], flour beetles [35], cockroaches [36], ants [37], and mosquitoes [38].

Previously, EOs and also the main constituents of the geraniums and junipers have been shown to possess a degree of anti-mosquito activities against the laboratory strains of malaria vectors [38–42]. However, comparative studies on their anti-mosquito activities against a susceptible laboratory strain and a wild pyrethroid-resistant population of malaria vectors in *An. gambiae* complex are missing. Therefore, the present study attempted to evaluate the mosquitocidal activities of EOs from *Juniperus virginiana* (JVO), *Pelargonium roseum* (PRO), and the main constituents of PRO against a laboratory colony of *An. gambiae s.s*. and a wild resistant population of *An. arabiensis*.

## Methods

### Plant materials and preparation of EOs

The aerial green parts of the *Juniperus virginiana* (Cupressaceae) and *Pelargonium roseum* (Geraniaceae) were respectively collected from green areas in the Production and Research Complex of the Institut Pasteur of Iran (51° 3′ 44′′ N, 35° 45′ 49′′ E, and 1330 m above sea level) and from a herb garden in Kashan (33.9850° N, 51.4100° E, and 900 m above sea level) during the summer 2019. The collected herbals were authenticated by Prof. Valiollah Mozaffarian, Research Institute of Forests and Rangelands, Tehran, Iran. After collection, plant samples were thoroughly washed twice with distilled water. Then 200 g of each plant material was transferred to a round bottom balloon (4000 mL) and dipped in 2000 mL of distilled water. The hydro-distillation process was carried out to extract the essential oils (JVO and PRO) using a Clevenger apparatus within a continuous extraction for 2 h [38]. The EOs were separated from water by decantation, dried over anhydrous sodium sulfate and stored in a dark glass vial at 4 °C until analysis.

## Chemical analyses of EOs

Chemical constituents of the EOs were characterized by gas chromatography-mass spectrometry (GC-MS) and achieved on a GC (HP 6890, Agilent, USA) equipped with a quadrupole mass spectrometer analyzer (HP 5973, Agilent). The MS was operated in an ionization voltage of 70 eV and an interface temperature of 280 °C. The MS ion source temperature and the MS quadrupole temperature were kept at 230 °C and 150 °C, respectively. Subsequently, 0.1 µL of the diluted sample was injected by an autosampler using a 100:1 split ratio and the inlet temperature of 280 °C. The sample was analysed on an open tubular capillary column (TRB-5MS, 30 m, 250 μm, and 0.25 μm). Helium (99.9995% pure) with a flow rate of 1 mL/min^− 1^ was the carrier gas. The sample was evaluated under the following settings: initial oven temperature at 36 °C for 5 min, ramp-up at 4 °C min^− 1^ to 200 °C and continued for 8 min, then increased to 280 °C with a ramp-up of 40 °C min^− 1^ for 10 min and overall run time of 66 min. ChemStation software was applied to assess chromatographic analytical data. Compounds were determined by comparing mass spectra with the Wiley7n.1. The Kovats retention index was calculated using an alkane standard mixture (C9-C24) based on the following formula: Kovats retention index = 100 × [n + (Tu-Tn)/(TN-Tn)], where n = the number of carbons in the alkane prior compound; Tu = the retention time of known compound; Tn = the retention time of the prior alkane; TN = the retention time of the next alkane.

The main chemical constituents of PRO, namely citronellol (CO; cat no. 27,470), geraniol (GO; cat no. 163,333), linalool (LO; cat no. L2602), L-menthone (MO; cat no. W266701), were procured from Sigma-Aldrich Company (Germany).

## Test organisms

Two sibling species of *An. gambiae* complex were investigated for the mosquitocidal properties of EOs in laboratory and semi-field conditions. The first species was *An. gambiae s.s.*, which has been maintained at the Tropical Pesticide Research Institute (TPRI) insectary since 1992 (*An. Gambiae s.s.* Kisumu strain). This sibling was reared routinely according to the modified MR4 protocols in the organized set up (28 ± 2 °C, 78 ± 2% relative humidity, and 12:12 light/dark photoperiodicity) [42, 43]. Adult mosquitoes were maintained on 10% sugar solution soaked on cotton wools. Female mosquitoes received blood meals from shaved rabbits to produce eggs every 3 days. The eggs were collected on a filter paper and left in a desiccator for maturation (48 h). The eggs were then floated in dechlorinated tap water for hatching. The larvae were fed with TetraMin Tropical Flakes fish food and maintained at the same conditions mentioned before. Third/fourth instar larvae were used in larvicidal assays. Larvae developed to pupal stage were collected using droppers and kept in adult rearing cages to emerge adults [43].

The second species, *An. arabiensis*, was collected from Mabogini rice irrigation schemes or from cowsheds in Lower Moshi, two malaria low-transmission area in the Kilimanjaro Region of Tanzania, using torch and mouth aspirator [44]. The gathered specimens were taken to the field insectary at TPRI and left for 24 h; during this time, they were provided with 10% glucose solution [44].

### **Larvicidal bioassays on*An. gambiae s.s.*in laboratory conditions**

**Fig. 1:**
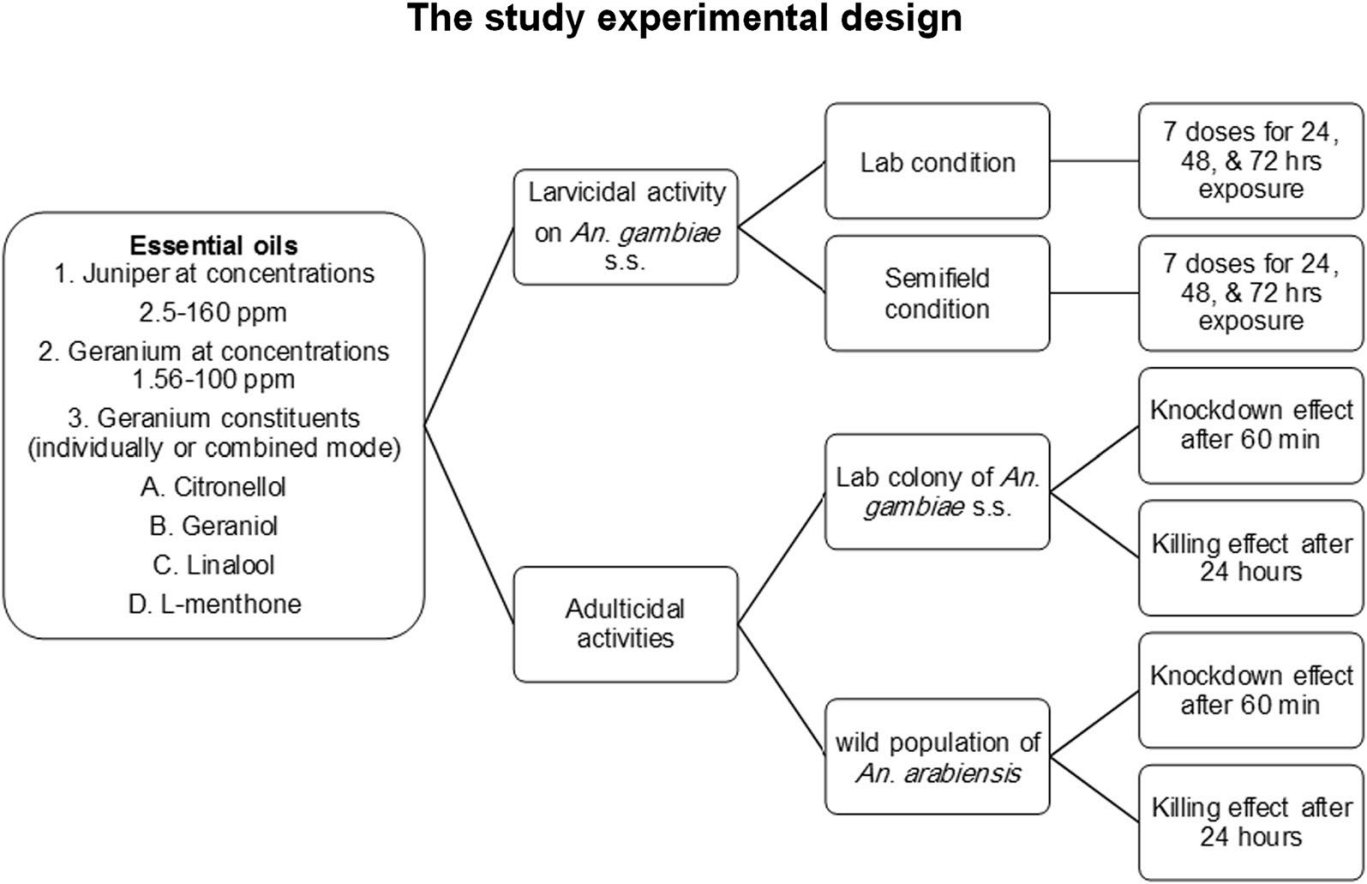
The schematic diagram illustrating the experimental design of the study

Prior to the onset of experiments, trial tests were accomplished in accordance with previously published results [38, 42], to identify the activity range of JVO, PRO, and the PRO constituents. Thereafter, each oil (1 ml) was added to ethanol 99% (9 ml) for the preparation of the stock solutions. The experiments were set with seven consecutive concentrations of the oils (2.5–160 ppm for JVO, 1.56–100 ppm for PRO, 1.56–100 ppm for main constituents of PRO, and a mixture of all components) by adding 1 mL of each concentration of the oils to a 250 mL glass beaker containing 99 mL of the dechlorinated water and 0.0007% Tween-80 [38]. The first control was normal tap water for breeding mosquitoes, while the second control was 1% ethanol containing Tween-80. Each concentration and control was repeated four times. Afterwards, 1 mL of each concentration of the under test oils was added to bowls to make up 100 mL of test solution. A minimum of 20 third/fourth instar mosquito larvae collected by a strainer with fine mesh was gently transferred to the bowls. The bioassays were performed in a test room (27 ± 2 °C and 70 ± 5% relative humidity). Larval mortality was recorded at 24, 48, and 72 h post exposure. Both dead and moribund larvae were recorded as dead. The experimental design of the study is depicted in a schematic diagram (Fig. 1).

### **Larvicidal bioassays on*An. gambiae s.s.*in semi-field conditions**

An experiment according to the protocol of the World Health Organization (WHO) [42], where seven concentrations for each JVO, PRO, and PRO constituents within 24 to 72 h was accessed for their larvicidal efficacy against *An. gambiae s.s*. vectors. The semi-field experiments were conducted in a screen-walled greenhouse according to Mdoe and others [45]. Bioassay was carried out in ambient conditions (temperature of ∼25–35 °C; relative humidity of ∼50–80%; 12:12 light/dark cycle). The experiments had seven concentrations for each of the test solution; each concentration had four replicates including two controls, one with normal rearing water and the other with 1% ethanol. Then 1 mL of each test concentration was added to bowls containing 99 ml of normal larval rearing water and 20 third/fourth instar larvae. Mortality was recorded at 24, 48, and 72 h post exposure. Both dead and moribund larvae were recorded as dead.

### **Adulticidal bioassays on*An. gambiae*complex in semi-field conditions**

To evaluate the susceptibility of adult *An. gambiae s.s.* and *An. arabiensis* mosquitoes, different concentrations of the oils (100 ppm for JVO, LO, MO, and MIX, 50 ppm for GO and CO, and 25 ppm for PRO) were dissolved in acetone and applied on filter papers. The procedure was carried out in line with a previously published protocol [46]. Briefly, by using a ruler, straight lines (with a two-cm line spacing) were drawn on filter papers (Ahlstrom Filter Paper, Grade 222, ca. number 2228 − 1416). The studied oils were impregnated on papers along straight lines using a micropipette in order that the oils spread uniformly on the filter papers. The control papers were impregnated with olive oil dissolved in acetone in the same way as experimental papers. Thereafter, impregnated papers were wrapped in an aluminum foil and stored in a refrigerator, ready for the experimental use. Each group of the oil impregnated papers was wrapped separately to avoid cross-contaminations.

Susceptibility tests of the adults from laboratory colony of *An. gambiae s.s.* and wild populations of *An. arabiensis* to filter papers impregnated with EOs (JVO, PRO, and the PRO constituents) were carried out according to WHO protocols [47, 48]. Clean white papers were inserted into six holding tubes and fastened with a steel spring wire clip. Twenty active female mosquitoes were aspirated (in batches) into six green dotted holding tubes, which were adjusted at an upright position for one hour. After this time, any damaged, dead, or knocked down mosquitoes were then replaced with healthy ones. Thereafter, another six tubes were prepared, four were separately marked with a red dot (exposure tubes), while two with a yellow dot (control tubes). Each of exposure tubes was covered with a filter paper impregnated with JVO, PRO, and the PRO constituents, whereas the remaining two tubes were impregnated with olive oil. The WHO standard permethrin (0.75%) impregnated papers were set as the positive controls.

Mosquitoes were gently blown into the exposure and control tubes, and then exposure tubes were detached and set in upright position [47, 48]. The insects were then kept in the tubes for 60 min, and their knock down rate was recorded. After 1 h, for recovery, the mosquitoes were transferred back to paper cups provided with 10% sugar solution soaked in cotton wool. After a 24-h recovery period, the number of dead mosquitoes were counted and recorded [45, 47]. Susceptibility testing results of EOs were interpreted based on WHO criteria [48]. Mosquitoes with the mortality rates of 98–100%, 90–97%, and 0–89% were classified as susceptible, tolerant and resistant groups, respectively. The status of the second group in terms of the existence of resistance was confirmed by conducting additional tests, as well as by determining the mortality rate of mosquitoes [48].

#### Data analysis

Mosquito larvae mortality data were subjected to Probit analysis to determine lethal concentrations (LC_50_ and LC_90_) of the larvae. The data were corrected by Abbott’s formula if mortality in control bowls ranged between 5 and 20% [38]. Mosquito adult mortality data were computed using one-way ANOVA analysis, followed by Tukey’s test. The insecticides resistance status in the *An. arabiensis* samples were determined based on the WHO protocol for the detection of insecticides resistance [48].

## Results

### Yields and chemical composition of the EOs

**Fig. 2:**
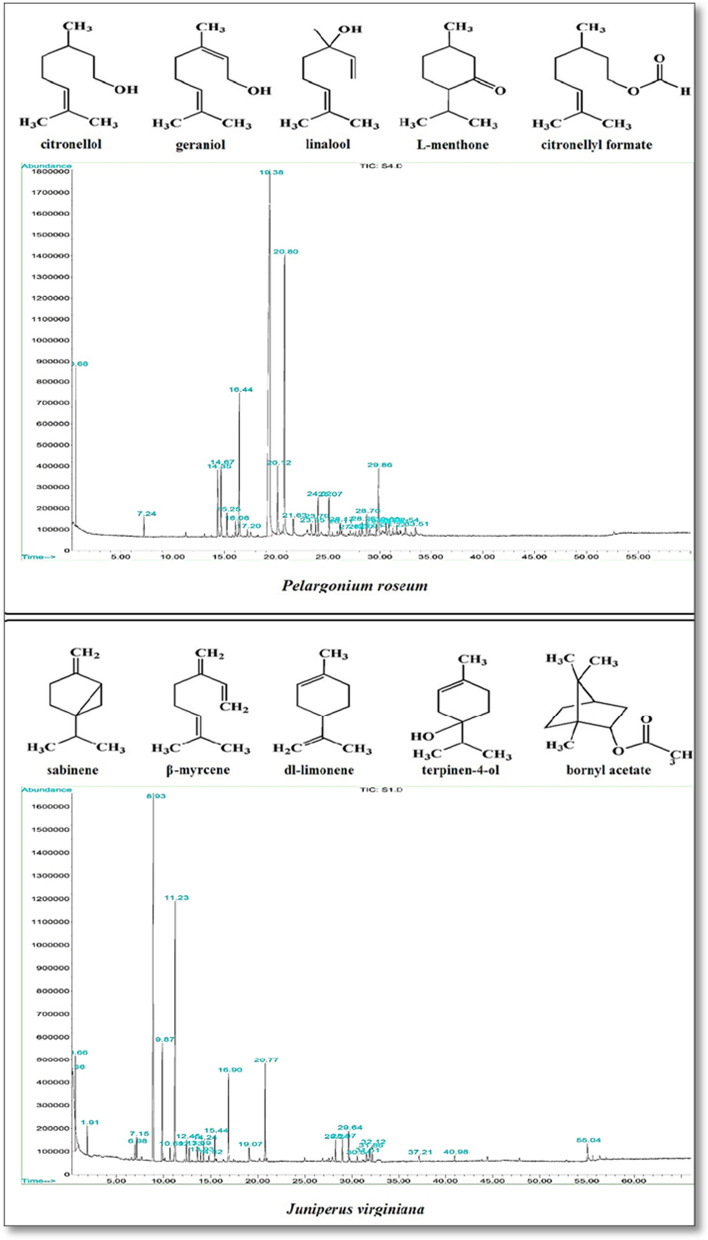
Chemical structure of the major constituents of *Pelargonium roseum* (citronellol, geraniol, linalool, L-menthone, and citronellyl formate) and *Juniperus virginiana* (sabinene, β-myrcene, dl-limonene, terpinen-4-ol, and bornyl acetate) essential oils along with their total ion chromatograms of GC-MS analysis

The hydrodistillation of the JVO and PRO green parts generated colorless and pale-yellow EOs of 0.75% and 0.38% (w/w) on fresh weight materials, respectively. The GC-MS analysis revealed the presence of 12 and 10 constituents in the JVO and PRO, corresponding to ~ 70% and ~ 83% of their total oils, respectively (Table 1). Five major components, comprising of sabinene (25.46%), dl-limonene (16.36%), β-myrcene (6.0%), bornyl acetate (5.18%), and terpinen-4-ol (4.90%), were identified with a similarity of ≥ 95% and quantity of > 4% for JVO (Table 1; Fig. 2). Also, five foremost components, i.e. CO (46.86%), citronellyl formate (15.78%), MO (6.57%), GO (4.34%), and LO (3.01%), at the resemblance of ≥ 94% and quantity of > 4% were identified for PRO (Table 1; Fig. 2).

**Table 1:** Chemical compositions of *Juniperus virginiana* and *Pelargonium roseum* essential oils identified by GC-MS

*Juniperus virginiana*					*Pelargonium roseum*				
Peak no.	Compounds	Kovats retention index	Quality (%)	Amount (%)	Peak no.	Compounds	Kovats retention index	Quality (%)	Amount (%)
2	1,3-bis(3-Phenoxy phenoxy) benzene	0.66	90	4.18	2	α-Pinene	930.30	97	0.85
4	α-Thujene	923.41	93	0.77	3	Linalool	**1099.11**	**97**	**3.01**
5	(-)-α-Pinene	927.94	97	1.15	4	cis-Rose oxide	1109.51	94	2.81
6	Sabinene	**969.30**	**97**	**25.46**	5	trans-Rose oxide	1128.31	91	1.04
7	β-Myrcene	**987.67**	**95**	**6.00**	7	L-Menthone	**1165.58**	**98**	**6.57**
9	dl-Limonene	**1020.02**	**99**	**16.36**	9	Citronellol	**1246.08**	**98**	**46.86**
10	γ-Terpinene	1053.42	97	1.13	11	Geraniol	**1264.34**	**94**	**4.34**
16	Camphor	1134.72	98	1.22	12	Citronellyl formate	1280.52	91	15.78
17	Terpinen-4-ol	**1179.11**	**96**	**4.90**	15	β-Bourbonene	1373.89	96	1.78
19	Bornyl acetate	**1279.82**	**98**	**5.18**	21	cis-Calamenene	1509.36	97	0.28
20	δ-Cadinene	1508.64	98	1.20	Total identified compounds = 83.32%				
22	Germacren D-4-ol	1557.60	98	1.56					
Total identified compounds = 69.11%									

The main components with similarities ≥ 94 and amounts > 3% are bolded

## Larvicidal activities of the oils in the laboratory and semi-field conditions

**Fig. 3:**
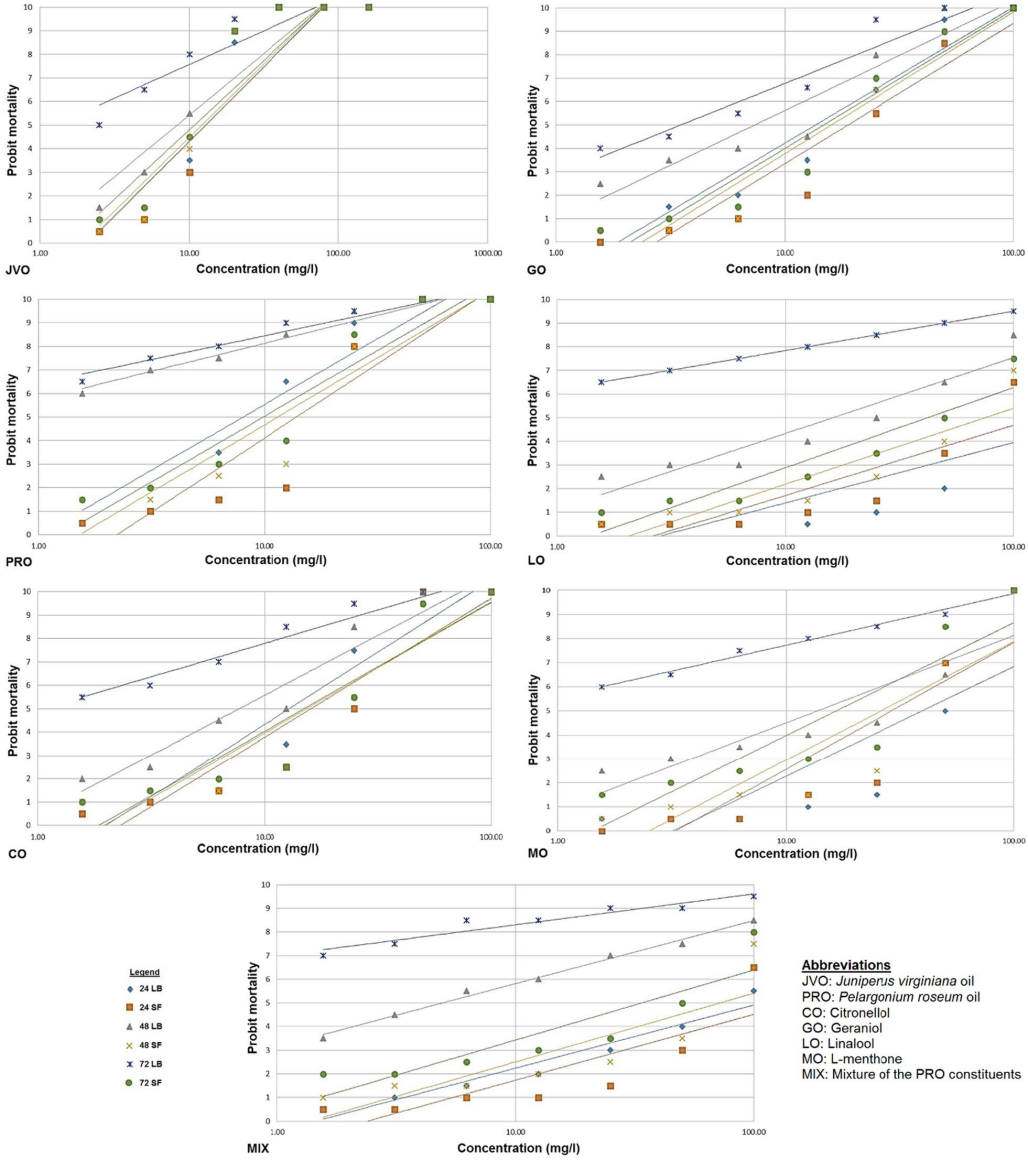
Probit regression line of larvae of *Anopheles gambiae s.s.* exposed to different concentrations of JVO, PRO, and the main components of PRO essential oils in laboratory and semi-field environments at times of 24–72 h

The trend in larvicidal activity (LC_50_/LC_90_) of the JVO, PRO, and the main constituents of PRO against the laboratory colony of *An. gambiae s.s.* in both laboratory and semi-field environments diminished with exposure time from 24 to 72 h (Table 2; Fig. 3).

**Table 2:** Toxicity of test compounds against the colony of *An. gambiae s.s.* in laboratory and semi-field conditions

Compounds		Environment	Lethal	Exposure times (24–72 h) LC_50_/LC_90_ (LCL-UCL) 95% CI (ppm)		
				24 h	48 h	72 h
JVO		LB	LC_50_	10.82 (8.61–13.68)	7.35 (5.55–9.54)	2.89 (1.36–4.33)
			LC_90_	24.96 (18.80-39.64)	21.52 (15.48–37.17)	13.46 (9.00-28.78)
		SF	LC_50_	10.75 (8.61–13.56)	10.11 (8.08–12.72)	9.06 (7.01–11.59)
			LC_90_	23.87 (18.09–37.63)	22.44 (17.03–35.35)	22.95 (16.95–37.58)
PRO		LB	LC_50_	7.13 (5.25–9.56)	1.26 (0.29–2.39)	0.90 (0.12–1.92)
			LC_90_	27.59 (18.83–50.32)	13.33 (7.80-37.03)	10.37 (5.94–29.17)
		SF	LC_50_	13.63 (7.19–28.36)	10.65 (5.03–23.61)	8.98 (4.93–16.23)
			LC_90_	43.06 (22.39-278.05)	46.39 (21.56-432.92)	39.34 (20.50-180.23)
PRO constituents	CO	LB	LC_50_	12.44 (9.49–16.49)	6.98 (4.89–9.67)	1.81 (0.67–3.03)
			LC_90_	41.43 (28.91–72.27)	35.31 (22.76–71.82)	15.12 (9.18–38.20)
		SF	LC_50_	16.29 (8.67–34.31)	15.28 (7.62–34.94)	14.79 (7.62–31.96)
			LC_90_	59.85 (29.79-406.81)	71.03 (32.02-681.41)	70.90 (32.60-571.30)
	GO	LB	LC_50_	13.43 (10.00-18.24)	6.76 (2.94–13.04)	3.64 (2.11–5.37)
			LC_90_	54.11 (36.05-102.03)	44.09 (20.40-341.20)	25.03 (15.41–58.48)
		SF	LC_50_	15.48 (11.59–20.98)	16.60 (12.60-22.17)	15.48 (11.59–20.98)
			LC_90_	59.76 (40.08-111.27)	57.18 (39.42-101.56)	59.76 (40.08-111.27)
	LO	LB	LC_50_	127.02 (43.87-136539.47)	16.16 (8.87–32.82)	0.48 (0.00-1.87)
			LC_90_	1451.18 (208.06-32552746815.41)	482.79 (145.11-8638.88)	48.79 (17.91-2121.94)
		SF	LC_50_	87.43 (49.95-249.19)	64.44 (37.47-167.69)	41.69 (24.57–98.25)
			LC_90_	887.87 (293.68-10638.56)	799.04 (262.91–9163.00)	682.13 (220.69-8071.05)
	MO	LB	LC_50_	39.52 (13.36-2934.82)	13.42 (4.40-48.54)	1.01 (0.08–2.51)
			LC_90_	183.90 (55.60-394844279.08)	248.41 (61.08-159509.43)	33.16 (15.44-222.24)
		SF	LC_50_	32.12 (15.81–93.08)	27.16 (13.12–88.32)	16.44 (6.55–57.53)
			LC_90_	99.89 (47.64-2206.37)	142.43 (54.61-4122.66)	127.80 (42.27-10660.87)
	MIX	LB	LC_50_	84.72 (42.27-377.75)	4.91 (1.42–9.73)	0.17 (0.00-1.14)
			LC_90_	1950.88 (418.83-107720.76)	268.76 (79.73-8325.05)	29.76 (10.44-3860.67)
		SF	LC_50_	97.53 (52.31-339.62)	65.01 (34.05-233.02)	34.10 (18.33–99.61)
			LC_90_	1235.35 (350.49-24425.89)	1485.44 (351.84-52350.81)	1163.09 (268.38-50690.93)

LC _50_ /LC 90 lethal concentration causing 50/90% mortality, *95% CI* confidence interval with a 95%, probability ppm: parts per million, *LB* laboratory conditions, *SF* semi-field conditions, *JVO* *Juniperus virginiana,* *PRO* *Pelargonium roseum,* *GO* Geraniol, *CO* Citronellol, *LO* Linalool, *MO* L-Menthone, *MIX* mixture of all four ingredients

The LC_50_/LC_90_ trends of JVO decreased with exposure times of 24, 48, and 72 h with the values of ≥ 2.89/13.46 and ≥ 9.06/22.44 ppm in laboratory and semi-field set-ups, respectively (Table 2). However, LC_90_ of semi-field (22.95 ppm) at the exposure time of 72 h showed slight increases (Table 2). The LC_50_/LC_90_ was lower in the laboratory compared to semi-field conditions throughout the exposure times (Table 2). JVO displayed a significant larvicidal activity against *An. gambiae s.s.* larvae with the LC_50_ values of 10.82 and 10.75 ppm at an exposure time of 24 h in laboratory and semi-field environments, respectively (Table 2).

For PRO, the LC_50_ trend decreased with increasing exposure times as 7.13–0.90 and 13.63–8.98 ppm for both laboratory and semi-field set-ups, respectively, while the LC_90_ trend reduced only in laboratory set-up with the values of 27.59–10.37 ppm. In the semi-field set-up, this trend enhanced from 43.06 ppm to 46.39 ppm with an increase in exposure time at 24 and 48 h (Table 2). However, throughout the exposure time, the LC_50_/LC_90_ was lower in the laboratory set-ups relative to semi-field one (Table 2).

In case of the PRO components, the LC_50_/LC_90_ trends decreased with increasing exposure times in laboratory conditions, with the range of 3.64−0.48/48.79–15.12 ppm after 72 h, while these values fluctuated with increasing times at the semi-field set-ups (Table 2). For all the PRO constituents, except for MO, the LC_50_/LC_90_ values were lower in laboratory set-ups as compared to those of semi-field ones (Table 2). The evaluation of a mixture of the main PRO constituents (MIX) under laboratory and semi-field conditions displayed a reduction in LC_50_ with increasing exposure time; in addition, an intense decrease was observed in laboratory but not semi-field conditions, with LC_50_ range of 84.72−0.17 and 97.53–34.10 ppm, respectively (Table 2). On the other hand, the trend of LC_90_ showed a reduction in a time-dependent manner, only for the laboratory conditions with LC_90_ range of 1950.88–29.76 ppm. Under semi-field conditions, LC_90_ increased at 48 h of exposure time and finally decreased after 72 h (Table 2). Also, a sharp decrease was found in laboratory set-ups when compared to semi-field ones (Table 2).

PRO and its major constituents (GO, CO, LO, MO, and MIX) indicated a significant larvicidal activity against the laboratory colony of *An. gambiae s.s.* in both laboratory and semi-field environments (Table 2). The crude oils of PRO had the most significant larvicidal activity relative to its respective constituents in the laboratory and semi-field environments, with the LC_50_ values of 7.13 ppm and 13.63 ppm at the exposure time of 24 h, 1.26 ppm and 10.65 ppm at the exposure time of 48 h, and 0.90 ppm and 8.98 ppm at the exposure time of 72 h.

In the laboratory environment and at the exposure time of 24 h, the most significant larvicidal activity among the PRO major constituents was reported for CO (12.44 ppm), followed by GO (13.43 ppm), MO (39.52 ppm), MIX (84.72 ppm), and finally LO (127.02 ppm) (Table 2). At the exposure time of 48 h, significant larvicidal activity was observed in MIX (4.91 ppm), followed by GO (6.67 ppm), CO (6.98 ppm), MO (13.42 ppm), and LO (16.16 ppm). In contrast, at 72-h exposure time, significant larvicidal activity was found in MIX with LC_50_ value of 0.17 ppm, followed by LO (0.48 ppm), MO (1.01 ppm), CO (1.81 ppm), and GO (3.64 ppm).

In the semi-field environment, significant larvicidal activity was first observed for GO with LC_50_ value of 15.48 ppm after exposure time of 24 h and then observed for CO (16.29 ppm), MO (32.12 ppm), LO (87.43 ppm), and MIX (97.53 ppm). However, at the exposure time of 48 h, CO had significant larvicidal activity with LC_50_ value of 15.28 ppm, followed by GO, MO, LO, and MIX (16.60, 27.16, 64.44, and 65.01 ppm, respectively). Considerable larvicidal activity, at the exposure time of 72 h, was identified for CO (14.79 ppm), followed by GO (15.48 ppm), MO (16.44 ppm), MIX (34.10 pm), and LO (41.69 ppm).

## Adulticidal activities of the oils in the semi-field conditions

**Fig. 4:**
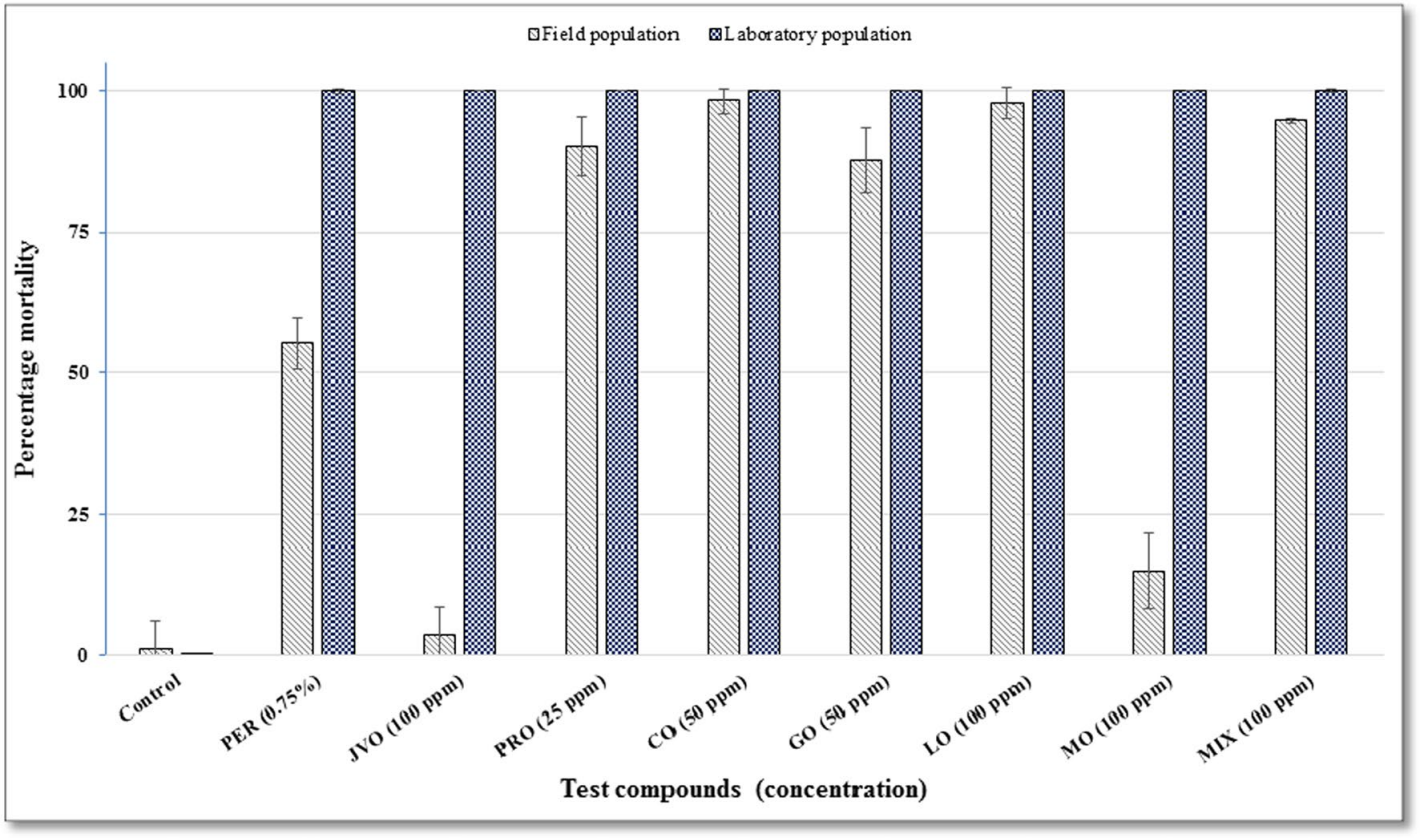
Adult percentage mortality post exposure in different oils (*Juniperus virginiana*, *Pelargonium roseum*, and the main constituents of *P. roseum*) for laboratory-reared and field populations of *Anopheles gambiae* complex.  *PER* permethrin, *JVO* *Juniperus virginiana,* *PRO* *Pelargonium roseum,* *CO* citronellol, *GO* geraniol, *LO* linalool, *MO* L-menthone *MIX* mixture of all four ingredients

The studied oils have displayed both knockdown and killing activities. The knockdown effects of all tested compounds for the laboratory colony of *An. gambiae s.s.* after 60 min of exposure was 100%. The knockdown effect was found to be 100% for field mosquitoes except for MO and JVO, which both had < 6% knockdown effect in the same time. However, the mortality rate effect of each EO tested was different. Following exposure to JVO-treated papers, the wild population of *An. arabiensis* exhibited the mean percentage mortality rate of 3.75%, whereas this rate was 100% for the susceptible laboratory colony of *An. gambiae s.s*., at the concentration of 100 ppm (Fig. 4). Nonetheless, in exposure to PRO-treated papers and at the concentration of 25 ppm, the wild population of *An. arabiensis* and susceptible laboratory colony of *An. gambiae s.s.* indicated the mortality rates of 90%, and 100%, respectively (Fig. 4).

Following exposure to CO-treated and GO-treated papers, 98.13% and 100% as well as 87.5% and 100% mean percentage mortality rates were obtained for the wild populations of *An. arabiensis* and laboratory strain of *An. gambiae*, respectively, at the concentration of 50 ppm (Fig. 4).

The wild population of *An. arabiensis* and susceptible laboratory colony of *An. gambiae s.s.* demonstrated the mean mortality rate of 97.81% and 100% as well as 15% and 100% when exposed to LO-treated and MO-treated papers, respectively, at the concentration of 100 ppm (Fig. 4). The exposure of the wild population of *An. arabiensis* to the mixture of PRO constituent-treated papers represented the mean percentage mortality rate of 94.69%, but that of the susceptible laboratory strain of *An. gambiae s.s.* showed to be 100%, at the concentration of 100 ppm (Fig. 4).

Following exposure to permethrin-treated papers (positive control), the wild population of *An. arabiensis* and laboratory colony of *An. gambiae s.s.* indicated the mortality rates of 55.14% and 100%, respectively, at the similar concentration of 0.75% (Fig. 4). The mean percentage mortality rates of the wild population of *An. arabiensis* and laboratory colony of *An. gambiae s.s.* exposed to olive oil-impregnated papers (negative control) were found to be 1.01% and 0.12%, respectively (Fig. 4); thus, there were no need for corrections via Abbott’s formula.

## Discussion

In this study, the studied oils (JVO, PRO, and PRO main components) showed anti-mosquito (larvicidal and adulticidal) activities under different conditions. The larval mortality rates were higher in laboratory environment than semi-field conditions, as reported by other studies [45, 46, 49, 50]. The trend of LC_50_ values of JVO larvicidal activity raised with increasing exposure time, which is in line with other investigations [51, 52]. In an Ethiopian study, similar variations have been reported in LC_50_/LC_90_ between the laboratory and semi-field environments [52]. This discrepancy in the results might be related to the semi-field environment that can decompose EOs into less or more toxic molecules [52]. The toxicity of PRO against *Anopheles* larvae in this study is in accordance with Tabari and others who evaluated the larvicidal properties of PRO and its major components against the larvae of *Culex pipiens* [41]. However, contrary to the results of this study, they found that combining all EO components produced higher larval mortality than any of the components alone [40]. This divergence between the results may be due to the fact that only 83.32% of total PRO oils in this study were identified or the anti-insect effects of only four major constituents of the PRO, including CO, MO, LO, and GO, were investigated. The diverse bioactivities (e.g. the excito-repellency and larvicide) of PRO and its main constituents against *Anopheles stephensi* have also been assessed by various research groups [42, 53–55]. It has been shown that the major components of PRO mentioned above are effective against two biotypes of *An. stephensi* (*mysorensis* and *intermediate*) in different doses [42]. It can be attributed to the lipophilic nature of EOs and accordingly its quick penetration into the larval body through the cuticle or ingestion [56]. The observations of this study also confirmed the rapid mortality of the larvae after exposure to the studied oils (unpublished data), which may reveal a neurotoxic mode of action, as noticed by other scientists, though calls for future detailed surveys [56, 57].

The EOs showed higher knockdown and mortality outcomes against the adults of *An. gambiae s.s.* than *An. arabiensis*. This finding could also be ascribed to the fact that plant-based natural products are readily photo-biodegradable and lead to secondary metabolites that might increase or decrease the compound toxicity. The JVO and PRO presented much higher knockdown effect than the WHO standard permethrin (0.75%) impregnated papers. Comparable results have also been achieved by Tabari and associates who explored moderate knockdown activity of CO, GO, and LO from PRO against *Cx. pipiens* adults [40]. Conversely, the present study reported that PRO and its constituents had high knockdown effects against *An. arabiensis*. A similar knockdown effect was also detected in a wild population of resistant *An. arabiensis* after exposure to the derived EOs of *Schinus terebinthifolia* [46]. However, its findings are different from that of the present study because the current study had presented the percentage of knockdown stage while the previous reported as overall outcome. This disparity could be due to difference in plant species from which the EOs were extracted or due to the different concentration of EOs used in both studies [40, 46]. The anti-insect mechanism of various toxins can be related to physiological or behavioral functions of insects. Some oils can affect the insect’s nervous system by antagonizing octopamine receptors or by inhibiting acetylcholinesterase at various stages of life history [58, 59]. In adult insects, volatile oils can disrupt the behavioral functions of antennal sensilla [60]. The effect of EOs against gut microbiota of insects can be considered as a new anti-insect mechanism [42]. Antibacterial activity of PRO against the intestinal bacterial flora of *An. stephensi* strains was assessed, and the result approved the importance of bacteria inhibition in insect’s survival [42, 61].

The mortality rates of adults from the laboratory strain of *An. gambiae s.s.* and wild populations of *An. arabiensis* exposed to JVO and PRO varied from 3.75 to 100%. Among all tested oils, JVO displayed the lowest mosquitocidal activity against the wild population of *An. arabiensis*. Parallel findings were also reported by a previous investigation that evaluated the toxicology of EOs from *Cupressus funebris*, *Juniperus communis*, and *Juniperus chinensis* (Cupressaceae) against *Aedes aegypti* [62]. On the other hand, the exposure of mosquitoes to PRO resulted in mortality, ranging from 90 to 100% at the concentration of 25 ppm. This mosquitocidal activity of PRO in this study is in conformity with the finding of a former survey, which recorded the mortality rate of 46% against adult *Cx. pipiens* at the concentration of 5 µg/l [41]. However, the adult mortality rate in the present study was twice as high as that reported in the study of Tabari [41]. Mosquito population in Lower Moshi is frequently subjected to insecticides due to agricultural activities [63, 64]; as a result, *An. arabiensis* may have developed knockdown resistance mutation against the insecticides [65]. Such dissimilarity in mortality rates could be explained by difference in mosquito and plant species under study. The findings of this research are in accord with those reported by Mbepera et al. on the resistance of *An. arabiensis* to insecticides recommended by the WHO for controlling malaria, including permethrin [63].

Of the constituents of PRO, CO showed the highest mosquitocidal activity against *An. arabiensis* with the mean percentage mortality rate of 98.13%, followed by LO and GO with 97.81% and 87.5% mortality rate, respectively. The lowest mosquitocidal activity in this species was detected in MO with the mean percentage mortality rate of 15%. However, the mixture of four PRO constituents demonstrated the mortality rate of 94.69% against *An. arabiensis*.

PRO presented the mean percentage mortality rate of 90% at low concentration of 25 ppm, while the effective components at concentration ≥ 50 ppm showed the mortality rates ≥ 87.5%. Increased activity of PRO at lower concentration may be related to the synergistic effect of CO, GO, and LO in crude oil and also other non-studied constituents of PRO, such as citronellyl acetate, cis-rose oxide, and β-bourbonene with the quantities of 15.78%, 2.81%, and 1.78%, respectively (Table 1). The mortality rate of 94.6% of the mixture of PRO constituents at high concentration of 100 ppm somehow confirmed the above conclusion.

Following exposure to permethrin as a positive control, the wild *An. arabiensis* population demonstrated the mean percentage mortality rate of 55.14%, but this rate was 100% for the susceptible laboratory colony of *An. gambiae s.s.* The mortality rates exhibited by the PRO constituents against *An. arabiensis* were higher when compared to the positive controls, except for MO (Fig. 4). Similar low mortality has also been found against permethrin in the wild populations of *An. gambiae s.s*. and *An. arabiensis* [8, 66].

Both PRO and JVO were stable under the laboratory and semi-field conditions where the anti-mosquito assays of this study were performed; nonetheless, the effectiveness of EOs could significantly be influenced by environmental factors such as ambient temperature. This key point has been addressed in the study of Pavela and Sedlák [67]. They discovered that the lethal effects of *Thymus vulgaris* EO against *Spodoptera littoralis*/*Culex quinquefasciatus* larvae were considerably influenced by temperature fluctuations [67]. Therefore, the effects of temperature on the bioassay of oils against *An. gambiae s.s*., which are often distributed in tropical regions, appeals for further investigation.

In the current study, the major components of two EOs were found to be sabinene (25.46%), dl-limonene (16.36%), β-myrcene (6.00%), bornyl acetate (5.18%), and terpinen-4-ol (4.90%) in JVO, while CO (46.86%), citronellyl formate (15.78), MO (6.57%), LO (6.02%), and GO (4.34%) were identified in PRO with a quality of ≥ 94% and quantity of > 4%. These observations were in agreement with earlier studies showing that the same compounds with varied quantities have anti-mosquito activities against the various biological forms of *An. stephensi* [68–70]. The acyclic monoterpenols, CO, GO, and LO are the structural analogs of each other [71]. GO can partly transform into LO by acid catalysis.

Metabolic biotransformation of GO into CO also occurs in many plants via ionization-dependent reaction or microbiological reduction [72, 73]. Lipophilic GO is used in transdermal drug delivery systems as a penetration enhancer [74]. CO causes the disruption of membrane integrity by inducing free radical generation [75]. In a previous study, the best result was achieved for CO with 100% mortality against various biological forms of *An. stephensi* larvae [42]. CO and GO have formerly suggested high toxicity against *Cx. pipiens* and *Pediculus humanus capitis* [41, 76], but LO indicated a weak toxicity against these strains [40, 41, 76]. Likewise, PRO exhibited larvicidal activities against *Cx. quinquefasciatus* [77], *Cx. pipiens* [41], and *Ae. aegypti* [52, 78], as well as *Anopheles* spp.

In the present study, the best larvicidal activity in laboratory conditions was related to PRO with LC_50_ value of 7.13 ppm after exposure time of 24 h. In the same conditions (LB, 24 h), JVO displayed the best LC_90_ (24.96 ppm). In the semi-field conditions, JVO with LC_50_/LC_90_ values of 10.75/23.87 ppm was also the best studied oil after exposure time of 24 h. Surprisingly, the MIX and LO showed weak results in laboratory conditions after 24 h (LC_50_ = 84.72, and 127.02 ppm, respectively), but after exposure time of 72 h, it displayed the best results (LC_50_ = 0.17, and 0.48 ppm, respectively). The comparison of LC_50_/LC_90_ presented 9.06/22.95 ppm in semi-field conditions for JVO and 0.90/10.37 ppm in laboratory conditions for PRO. These results confirm the potent larvicidal activity of PRO and JVO and their appropriate application in mosquito control programmes.

Before the development of synthetic insecticides, integrative control with a strong focus on larvae sites (mostly mechanical/physical control) were the most common and efficient methods [79]. Botanicals used to combat insects from a long time ago also gained high popularity in the old communities [80]. The beginning of the insecticide revolution of the 1940−1950s led to the large-scale use of chemical insecticides that during future years resulted in various health and environmental problems. Recently, the evaluation of the susceptibility and irritability level of insects to different insecticides confirmed the decreased efficiency of most of these agents [66, 79, 81]. Scientific community and general public now consider herbal formulations as the most appropriate and safest option in killing and repelling mosquitoes due to the problems of chemical pesticides. Increasing demands for these natural products verify the importance of the evaluation and development of new botanical mosquitocides/repellents [82, 83].

Thanks to their chemical composition, JVO and PRO possess numerous biological activities of great interest in food and cosmetic industries, as well as in the human health field. The oil extracted from JVO, with LD_50_ oral (rat) > 5000 mg/kg body weight and with LD_50_ dermal (rabbit) > 5000 mg/kg body weight can be considered nontoxic [84]. Moreover, PRO has not shown any toxic effect on rats with LD_50_ > 5000 mg/kg body weight via oral gavages and on rabbits with LD_50_ > 2000 mg/kg body weight via topical application [85]. However, the efficiency of these plant-based natural products affirmed toxic effects on various arthropods (Additional file 1: Table S1) [86–95].

As highly volatile compounds are found in JVO and PRO, these oils are typically impossible to leave residues in food or the ecosystems. Consequently, the real concept of green pesticides can easily be attributed to these oils. However, the water solubility and spreading capacity, as well as the persistence of the oils need to be improved through nanoemulsion-based delivery systems, as discussed in the literature [42]. Additional assays are required to scrutinize the outcome of sublethal concentrations of the oils on life history parameters of the non-target organisms and also the designation of their LD_50_ values in warm-blooded vertebrates.

## Conclusions

The present study confirms the anti-mosquito effects of potential eco-friendly EOs on the dominant malaria vectors in Africa for the first time. The findings of this study reflect that the studied oils possess high anti-mosquito properties in terms of larvicidal, knockdown, and mortality when used against the susceptible laboratory and resistant wild populations of *An. gambiae s.l.* The rapid actions of these oils against different life stages of the highly efficient malaria vectors presumably disclose their neurotoxic activity. As a result, these oils have the potential for the development of new, efficient, safe, and affordable agents for mosquito control in the field condition. However, further investigations are necessary to improve their performance, by the use of nanoformulations techniques that produce high-penetrating and slow-releasing products or by the combination of EOs with other mosquitocides.

## Supplementary Information

**Additional file 1**: **Table S1. **Various activities of *Pelargonium roseum* and *Juniperus virginiana* essential oils against arthropods.

## Data Availability

The datasets supporting the conclusions of this article are included within the article and its additional file.

## Abbreviations

CO: Citronellol
EOs: Essential oils
GO: Geraniol
Hrs: Hours
JVO: *Juniperus virginiana*
LB: Laboratory condition
LC: Lethal concentration
LCL: Lower control limit
LO: Linalool
MO: L-Menthone
ppm: Parts per million
PRO: *Pelargonium roseum*
SF: Semi-field condition
TPRI: Tropical Pesticides Research Institute
UCL: Upper control limit
WHO: World Health Organization

## Declarations

### Note

The main components with similarities ≥ 94 and quantity > 3% are bolded.
