# Supplementary material for: Anti-mosquito properties of Pelargonium roseum (Geraniaceae) and Juniperus virginiana (Cupressaceae) essential oils against dominant malaria vectors in Africa
Source: Malar J. 2022 Jul 14;21:219. doi: 10.1186/s12936-022-04220-8 (PMC9284854; doi:10.1186/s12936-022-04220-8)
Supplement: Supplementary file 1 — Additional file 1: Table S1. Various activities of Pelargonium roseum and Juniperus virginiana essential oils against arthropods. [file 12936_2022_4220_MOESM1_ESM.docx]

**Table S1: Various activities of *Pelargonium roseum* and *Juniperus virginiana* essential oils against arthropods**

| ***Pelargonium roseum* essential oil** | | | | ***Juniperus virginiana* essential oil** | | | |
| --- | --- | --- | --- | --- | --- | --- | --- |
| **Target species** | **Biological activity** | **Major constituents (abundance %)** | **Reference** | **Target species** | **Biological activity** | **Major constituents**  **(abundance %)** | **Reference** |
| *An. stephensi* (Mysorensi and  Intermediate)  (Diptera: Culicidae) | Larvicidal (LC_50_ =11.5 & 12.5 ppm, respectively) | Citronellol (21.34), L-menthone (6.41), linalool (4.22), Geraniol (2.19) | [42] | *An. stephensi* | Larvicidal (LC_50_ =11.693 ppm); Pupicidal (LC_50_ =9.640 ppm); Repellency (54.63% protection & 45.37% mortality) | Terpinen-4-ol (25.21), camphor (19.89), E-3-hexen-1-ol (13.30), γ-terpinene (7.86), and L-menthone (2.27) | [38] |
| *S. exigua* (Lepidoptera: Noctuidae) | Larvicidal (90% mortality); Adulticidal (40% mortality); Fecundity (eggs/female, week) | Geraniol (13.56); menthol-acetate (10.37); (E)-β-caryophyllene (7.55) | [86] | *S. exigua* (Lepidoptera: Noctuidae) | Larvicidal (92% mortality); Adulticidal (0% mortality); Larvae hatchability (70.04 %) | Cis-thujopsene (37.92); cedrol (34.96); α-tedrene (6.36); γ-himachalene (5.02); longipinene (3.14) | [86] |
| *Cx. pipiens*  (Diptera: Culicidae) | Larvicidal (LC_50_ =5.49 μg/mL) | β-Citronellol (35.9); geraniol (18.5); Linalool (5.72) | [41] | *Musca domestica*  (Diptera: Muscidae) | Insecticidal (89% mortality) | --- | [93] |
| *An. stephensi* (Diptera: Culicidae) | Repellency (77% exit trap  rate); (3% blood feeding rate) | --- | [53] | *Camptomyia*  *corticalis* (Diptera: Cecidomyiidae) | Fumigant toxicity (LC_50_ = 0.9 mg/ml) | --- | [94] |
| *Amblyomma americanum*  (Ixodida: [Ixodidae](https://www.google.com/search?rlz=1C1SNNT_enIR916IR916&sxsrf=ALeKk035fCTiAhwsStmD47or_osbYlO7cw:1616352220139&q=Ixodidae&stick=H4sIAAAAAAAAAONgVuLSz9U3sMw2N7QwWMTK4VmRn5KZkpgKAGODVWIZAAAA&sa=X&ved=2ahUKEwiw4KafhcLvAhXMOcAKHdjGAsYQmxMoATAmegQILRAD)) | Repellency (>90%) | Citronellol (27); geraniol (11); citronellyl formate (7); 10-epi-γ-Eudesmol (6) | [87] | *Ae. aegypti* (Diptera: Culicidae) | Larvicidal (LC_50_ ~1 ppm) | --- | [51] |
| *Tetranychus urticae* (Acari: Tetranychidae) | Adult Fumigant (94% mortality); Egg hatchability Fumigant (92.4% mortality) | --- | [88] | *An. stephensi*  (Diptera: Culicidae) | Larvicidal (LC_50_ <10 ppm) | --- | [51] |
| *Cx. quinquefasciatus*  (Diptera: Culicidae) | Larvicidal (LC_50_ =130.30 μg/mL) | --- | [89] | *Cx.*  *quinquefasciatus* (Diptera: Culicidae) | Larvicidal (LC_50_ <10 ppm) | --- | [51] |
| *Rhipicephalus (Boophilus)* *annulatus*  (Acari: [Ixodidae](https://www.google.com/search?rlz=1C1SNNT_enIR916IR916&sxsrf=ALeKk035fCTiAhwsStmD47or_osbYlO7cw:1616352220139&q=Ixodidae&stick=H4sIAAAAAAAAAONgVuLSz9U3sMw2N7QwWMTK4VmRn5KZkpgKAGODVWIZAAAA&sa=X&ved=2ahUKEwiw4KafhcLvAhXMOcAKHdjGAsYQmxMoATAmegQILRAD)) | Acaricidal (98.3% mortality); (87.5% egg laying failure) | β-Citronellol (47.46); citronellyl formate (11.00); geraniol (8.74); iso-Menthone (4.32); linalool (3.07) | [90] | *Ae. aegypti* (Diptera: Culicidae) | Repellency (37.8%); Protection period (180 min); Landing (8%); Biting (1.2%) | --- | [78] |
| *Trialeurodes vaporariorum*  (Homoptera: Aleyrodidae) | Adulticidal (92% mortality); Nymphicidal (87% mortality); Ovicidal (88% mortality) | --- | [91] | *An. stephensi*  (Diptera: Culicidae) | Repellency (38.1%); Protection period (480 min); Landing (5.2%); Biting (0.0%) | --- | [78] |
| *Ixodes*  *ricinus* (Acari: Ixodidae) | Strong repellency (86%); Nymphs  Attracted (0%) | --- | [92] | *Cx.*  *quinquefasciatus* (Diptera: Culicidae) | Repellency (100%); Protection period (480 min); Landing (0.0%); Biting (0.0%) | --- | [78] |
| *An. stephensi*  (Diptera: Culicidae) | Repellency (61.9%); Protection period (480 min); Landing (2.8%); Biting (0.4%) | --- | [78] | *Ae. Aegypti*  (Diptera: Culicidae) | Larvicidal (100% mortality) | --- | [95] |
| *Cx.*  *quinquefasciatus* (Diptera: Culicidae) | Repellency (100%); Protection period (480 min); Landing (0.0%); Biting (0.0%) | --- | [78] | *Cx. pipiens pallens*  (Diptera: Culicidae) | Larvicidal (100% mortality) | --- | [95] |
| *Ae. aegypti* (Diptera: Culicidae) | Repellency (78.4%); Protection period (150 min); Landing (2.4%); Biting (0.8%); Larvicidal (73.3% mortality) | --- | [51, 78] |  |  |  |  |
